# Turning over New Leaves

**Published:** 1888-07-28

**Authors:** 


### TURNING OVER NEW LEAVES.

[Books for Review should be sent to The Editor, The Lodge, Porchester Square, W.]

### A Socialist Novel.*

MISS HOWELL'S story escapes in great measure the reproach that belongs to propagandist novels, because it does not pretend to describe the possible future successes of the cause she has at heart, but only depicts its present difficulties. The story is divided into two books. The first deals with the hero's mother, who passes from a formal adhesion to Christianity to theism, and thence to agnosticism and atheism, finding peace only in the last. The second shows the hero finding in socialism "a more excellent way" of life. In both sections there is undeniable truth and earnestness; in both the writer's aspirations somewhat mar the justice and integrity of her insight. It is true, most bitterly true—and it tells against the progress of Christianity as much as against the progress of atheism—that men who are freethinkers themselves insist on their wives maintaining the forms of religious belief and bringing up their children in them, because "religion is a social question," and to give up the semblance of it involves certain social disabilities. It is true also that there is an illogical notion prevalent that religion is more necessary for women and the so-called "lower classes" than for intelligent men, who can guide their way in all—at least apparent and worldly—uprightness without it. It is true also that women often do, like Agatha Hathaway, pretend a conformity they do not feel for fear of injury to their position and loss to their affections. But surely it is wrong to depict such a woman as in any degree heroic. "Biding one's time" is prudent, but not usually noble, and Mrs. Hathaway, going to services she believes to be superstitious and degrading, for fear her son should be parted from her, and saying nothing of her own beliefs till his worldly welfare is secured, offers an example of a very common kind of motherly love, but not the highest, which surely must seek truth as the supreme good, to be bought at any price. This materialism of aim is the flaw of the book. Otho Hathaway, the hero, is not an ignoble character; his aims are good and unselfish, but they do not go far enough. We do not despise the feeding of the hungry and the clothing of the naked, we admit that for the rich to enjoy luxury while the poor are starving is utterly wrong. We hold the socialist motto of freedom, equality and brotherhood to be a glorious one, but it must fail of all permanently beneficial effect as long as these qualities find their satisfaction in nothing more than equal shares in the earth and its produce. Let the body's needs be satiated, there remains the soul—intellect, æsthetic sense, call it what we will—the craving for something more than physical good, which remains as much in bondage to hunger and thirst as ever. Miss Howell seems to think that once the material equality is obtained all other things must follow. Selfishness, cruelty, greed, are all the result of physical conditions which socialism will remove, and we shall have "virtue served out with the soup at six," as Mrs. Browning sarcastically puts it. The interdependence of physical and moral conditions is undeniable—up to a certain point!—but the moral sense of a nation is not to be gauged by its material prosperity, nor do comfort and virtue inevitably increase in direct proportion. The brotherhood of man is something deeper than the sociability of guests at the same banquet, and must include other interchanges than those of bodily comforts—interchanges of help and hope in which equality is impossible. Socialism has high aims, but hitherto it has failed because it has sought to attain them by purely material means. We have no fault to find with Miss Howell's ambitions; they are pure and unselfish. But when she and those who think with her find that their well-fed proletarians are not after all better men in any true sense than the hungry ones, we would remind them of the wise words of the poet we have already quoted, who assuredly was not the less sage a thinker because she was a woman and a poet:

"A starved man
Exceeds a fat beast; we'll not barter, sir,
The beautiful for barley. And, even so,
I hold you will not compass your poor ends
Of barley-feeding and material ease,
Without a poet's individualism
To work your universal. It takes a soul
To move a body: it takes a high-souled man
To move the masses, even to a cleaner stye:
It takes the ideal to blow a hair's breadth off
The dust of the actual. Ah! your Fouriers failed
Because not poets enough to understand
That life develops from within."

We speak in no scorn of a book that shows both talent and honesty, but nevertheless in earnest protest against any scheme of reform that seeks to better man's physical condition without taking heed of the eternal cravings of his soul.

* "A More Excellent Way." By Constance Howell. Swan Sonnenschein, Lowry, and Co.

### Abortive Treatment of Specific Febrile Disorders.*

THE abortive treatment of all kinds of diseases which run a specific course has always had a great fascination for the medical mind. To watch a patient through a disease, warding off dangers, helping debility, and generally "doing the best you can" for him is one thing; the detection of the disease at its very outset, the prompt application of a specific remedy, the curing or cutting short of the disease in a few hours or days—this is something quite different, and much more to the taste of the medical enthusiast. But is it practicable? Is it often done; is it ever done? May we ever expect to do it systematically, constantly, and with unmistakable certainty? There are some who will answer "No" to all these questions. Dr. Illingworth will answer "Yes." The writer of this little book thinks he has found in biniodide of mercury a germicide, a specific poison for the villainous little creatures whose presence in the blood developes scarlatina, diphtheria, and other definite diseases with all their miserable train. If Dr. Illingworth really has discovered such a remedy as he thinks he has he deserves to take rank with Simpson, Jenner, and the most famous lights of medicine. But has he? That is the point. That the biniodide of mercury is of use in certain conditions of the throat in diphtheria and scarlet fever is one thing: That this remedy in non-poisonous doses is a germicide of such a character that it will at once and effectually destroy the germs of these diseases, and so prevent the diseases running their usual course is quite another. Dr. Illingworth's book is an honest record of honest and successful work in the practice of medicine. As such, it is well worth the perusal of all candid practitioners. This much we gladly admit. It may, too, be a scientifically demonstrable fact that the biniodide of mercury is such a germicide as Dr. Illingworth thinks it to be. But at any rate, the demonstration is still to be made. Dr. Illingworth has recorded an experience; but he started out to prove a case. As an experience his book is very valuable; but if he desires to prove his case, he must set to work anew, make his observations full and complete, eliminate sources of fallacy, arrange his facts, build up a sufficient induction, and then marshal his arguments in such a way that every man of scientific and candid mind shall be convinced of the soundness of his conclusions. If he can do this he will make an advance in practical medicine such as has not been witnessed since the estab-

* "The Abortive Treatment of Specific Febrile Disorders by the Biniodide of Mercury." By C. R. Illingworth, M.D. London: H. K. Lewis, Gower Street.

lishment on an immovable basis of the antiseptic system of treatment. Is he equal to the gigantic task? Is the task capable of being accomplished by any single man, or by the whole medical profession?

**Indian Diseases.***

THAT a second edition of Sir William Moore's work on the diseases of India has been called for is in no way remarkable, It is marked in a peculiar degree by thoroughness, honesty. and simplicity. The author states most frankly the opinions held by those who have preceded him in the investigation of the diseases peculiar to Hindostan, and while he gives his opinion fearlessly when it differs even from authorities of undoubted eminence, he is careful to lay before his readers the facts and observations, obtained under exceptionally advantageous circumstances, on which he bases them. Throughout the book the language is clear and concise, and the style has literary merits which medical writers do not always display. It is a book which the youngest student may read with almost entire comprehension, while an experienced practitioner may study it not only with interest but with profit. Of the diseases treated only a few, such as fungus foot, insolation or sunstroke, and moon-blindness, are peculiar to tropical climates, but Sir William Moore is careful to point out the differences which characterise the tropical developments of diseases familiar to Europe. He is very severe on the contradictory accounts of that mythical terror called malaria, which he takes to be simply another name for insanitary conditions of any kind; and his remarks on the nature and origin of such diseases as leprosy and scurvy are as interesting as they are rational.

**The Teachers' Bible.***

THIS edition of the Bible, neatly bound, beautifully printed, convenient in size and moderate in weight, should prove a treasure to all those who wish to give lessons in Scripture to the young. It contains in its notes and appendices a remarkable amount of that sort of information about the Jews, their country and history, our Lord and his disciples, which is so necessary to give freshness and vividness to the familiar words of the Bible, which have sometimes, even in the case of children, fallen on the ear so often that they have lost all effect of reality as a narrative of human life, and thereby been deprived of much of their power as a divine revelation.

